# High Grade Myofibroblastic Sarcoma of Paratesticular Soft Tissues

**DOI:** 10.1155/2014/768379

**Published:** 2014-08-11

**Authors:** Ioannis Anastasiou, Panagiotis K. Levis, Ioannis Katafigiotis, Georgios Karaolanis, Viktoria-Varvara Palla, Evangelos Felekouras, Antonios Athanasiou, Marina Perdiki, Dionysios Mitropoulos, Constantinos A. Constantinides

**Affiliations:** ^1^1st University Urology Clinic, Laiko Hospital, University of Athens, 17 Agiou Thoma Street, Attiki, 11527 Athens, Greece; ^2^Department of Urology, Athens University Medical School, Laiko Hospital, 17 Agiou Thoma Street, Attiki, 11527 Athens, Greece; ^3^2nd Department of Surgery, Laiko General Hospital, Medical School of Athens, 17 Agiou Thoma Street, Attiki, 11527 Athens, Greece; ^4^Department of Obstetrics and Gynecology, G. Gennimatas General Hospital, Mesogeion Avenue 154, Attiki, 11527 Athens, Greece; ^5^1st Department of Surgery, University of Athens Medical School, 17 Agiou Thoma Street, Attiki, 11527 Athens, Greece; ^6^Department of Pathology, National and Kapodistrian University of Athens, 17 Agiou Thoma Street, Attiki, 11527 Athens, Greece

## Abstract

Tumors of the paratesticular region most often arise from the soft tissue surrounding the spermatic cord and the epididymis or from the soft tissue (dartos muscle) of the scrotal wall. Paratesticular tumors, despite their rarity, present a high incidence of malignancy (30%), and the therapeutic approach of choice is surgical resection with negative margin. The grade, the histology type, the presence of metastases during the diagnosis, the size of the tumor, the age of the patients, and the surgical margins are all important prognostic factors. We present a case report of a 86-year-old patient with a high grade paratesticular and scrotum sarcoma of soft tissues which was presented as a hard painful mass of the scrotum. The patient was subjected to high ligation of the spermatic cord and received no further treatment and 6 months after the operation no local or systematic recurrence was observed.

## 1. Introduction

Soft tissue sarcomas (STSs) are a heterogeneous group of dense tumors which originate from embryonic mesenchymal cells (mesoderm), presenting multiple clinical patterns [1–4]. Respectively to the genitourinary (GU) tract, STSs are relatively rare tumors, accounting for 2.1% of STSs and 1% to 2% of all malignancies of the GU tract [2, 3]. Tumors of the paratesticular region most often arise from the soft tissue surrounding the spermatic cord and the epididymis or from the soft tissue (dartos muscle) of the scrotal wall [1, 5, 6]. Paratesticular tumors, despite their rarity, present a high incidence of malignancy (~30%), and the therapeutic approach of choice is surgical resection with negative margin [1–3, 7], while the benefits of adjuvant radio-/chemotherapy and/or retroperitoneal lymph node dissection (RPLND) are still under investigation [4, 8].

## 2. Case Report

**Figure 1:**
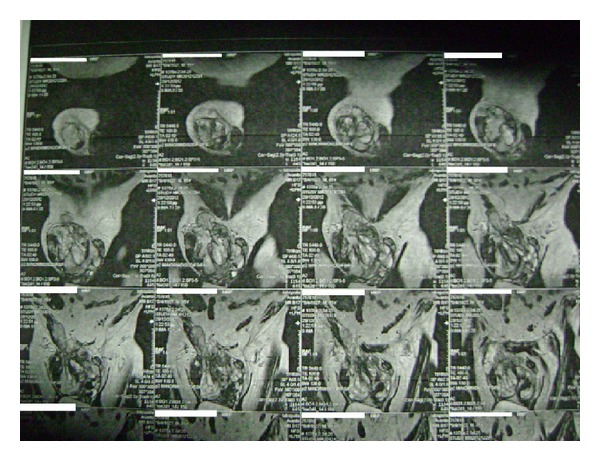
MRI of the pelvic depicting the paratesticular mass.

**Figure 2:**
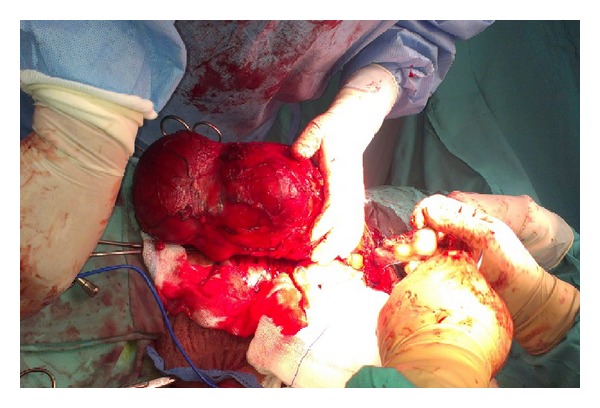
Macroscopic appearance of the huge paratesticular tumor.

**Figure 3:**
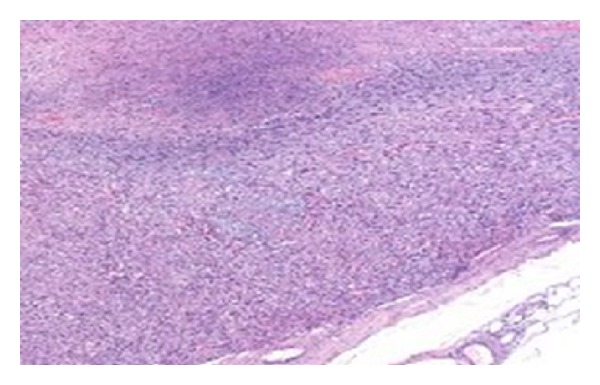
High grade (grade 2) sarcoma of paratesticular soft tissues with a myofibroblastic phenotype.

**Figure 4:**
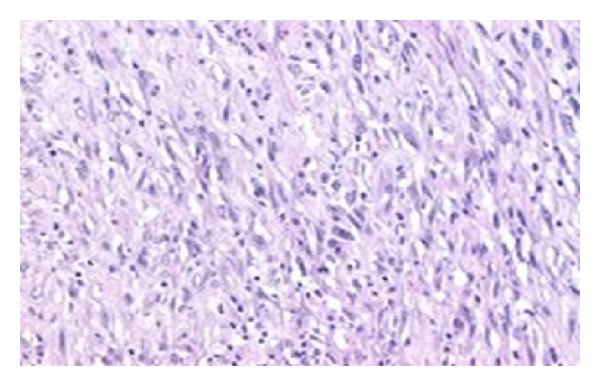
High grade (grade 2) sarcoma of paratesticular soft tissues with a myofibroblastic phenotype.

A 86-year-old male presented in the emergency room of the general surgery with pain and discomfort in the right iliac fossa reflecting to the ipsilateral scrotum/testis. His medical history reported a known incomplete descent of the right testis and a two-year small painful mass in the right iliac fossa, gradually expanding to the ipsilateral hemiscrotum, which was diagnosed earlier in another hospital as an inguinal hernia. Physical examination revealed a hard, mildly painful mass in the anatomical place of the right testicle, lying up until the iliac fossa where pain was more profound on palpation. No other constitutional sings were present and he had never received anabolic corticosteroids or radio therapy. His medical record revealed a history of heart failure (EF ≈ 30%) due to myocardial infraction (MI) prior to angioplasty (2005) and a second MI in 2009. Full blood count, lever function, serum glucose, urea, creatinine, LDH, and electrolytes levels revealed no abnormalities throughout his hospitalization. Patient's biomarkers, CEA, AFP, CA-19.9, and PSA, showed also no abnormalities. Unfortunately no *β*-HCG was measured preoperatively from the surgery department. The Magnetic resonance imaging (MRI) of the abdomen and the pelvic showed a huge mass in the scrotum arising from the inguinal canal (Figure 1). An exploratory operation of the inguinal canal was scheduled and during the operation the contribution of an urologist was asked. Finally a resection of the mass was performed with high ligation of the spermatic cord (Figure 2). The postoperative period was uneventful and the patient exited the hospital 2 days after the operation. The histology report referred to a macroscopic specimen consisting of a large tumor arising in right scrotum and encircling the testis, epididymis, and spermatic cord. Sections showed a high grade sarcoma (grade 2 according to Coindre et al.) of paratesticular soft tissues with a myofibroblastic phenotype [SMA (+) in a moderate number of cells, desmin (+) in isolated cells, h-caldesmon (−), S-100 (−), CD-34 (−), myogenin (−), and Ki-67 45%]. The neoplastic cells were fusiform with moderate nuclear atypia and moderate mitotic activity (11 mitosis/10 HPF) Figures 3 and 4. Few necrotic areas (<50%) and areas of stroma hyalinization were also seen. The neoplasm was widely invasive into the surrounding paratesticular soft tissues, without affecting the spermatic cord, the testis, and the epididymis and with negative surgical margins. The final diagnosis was that of a high grade (grade II, Coindre et al.) paratesticular and scrotum sarcoma of soft tissues.

## 3. Discussion

The majority of the masses occurring in the scrotal sac are within the testis and usually neoplastic [1]. The paratesticular region has a wide variety of epithelial, mesothelial, and mesenchymal elements and the neoplasms that can occur have also a wide variety of both benign and malignant patterns [1]. Soft tissue sarcomas of the genitourinary tract are rare but account for the most of the malignancies occurring in the paratesticular region [1, 2]. Because of the rarity of the soft tissue sarcomas data concerning these malignancies are sparse [2]. A definite diagnosis of a paratesticular tumor and distinction from a testicular tumor cannot be made preoperatively because both categories can present as a scrotal mass and swelling [1]. Complete surgical resection with high ligation of the spermatic cord is considered the best initial treatment [1, 3, 9]. Additional treatment with adjuvant chemotherapy, radiotherapy, and additional surgical resection may be necessary for control of the tumor [1, 3]. Due to the high local relapse rates (25–37%) in the scrotum and the groin after orchidectomy adjuvant locoregional radiotherapy and surgery may be needed in order to reduce the risk of local recurrence [1, 10, 11]. In our case we chose a radical resection with high ligation of the spermatic cord without an additional treatment due to the age and the comorbidities of the patient. During the first followup with clinical examination and a computed tomography of the thorax, the abdomen, and the pelvis six months after the operation no local or systematic recurrence was observed and a new followup is scheduled one year after the operation. It is also important to mention that a retroperitoneal lymph node dissection (RPLND) has been suggested as a treatment adjunct due to the high rates (14–29%) of nodal involvement of the paratesticular tumors but its role remains controversial [1, 11, 12]. One suggested approach concerning the RPLND is that in patients with high risk of nodal metastasis and subsequently high risk of systematic disease systematic treatment is more beneficial than local treatment while in patients with radiologically suspicious lymph nodes a RPLND is more appropriate [13–15]. The grade, the histology type, the presence of metastases during the diagnosis, the size of the tumor, the age of the patients, and the surgical margins are all important prognostic factors [1, 7]. Paratesticular tumors show a 58–80% overall 5-year disease specific survival while the high grade tumors show worst outcome with high rates (62%) of nodal or systematic failure [11, 14].
